# Engineering Metamaterials for Civil Infrastructure: From Acoustic Performance to Programmable Mechanical Responses

**DOI:** 10.3390/ma18174032

**Published:** 2025-08-28

**Authors:** Hao Wang, Shan Zhao, Chen Xu, Kai Sun, Runhua Fan

**Affiliations:** 1 College of Ocean Science and Engineering, Shanghai Maritime University, Shanghai 201306, China; 202330410162@stu.shmtu.edu.cn (H.W.); 17778790645@163.com (C.X.); kais@shmtu.edu.cn (K.S.); 2 College of Civil Engineering, Tongji University, Shanghai 200092, China

**Keywords:** metamaterials, acoustic performance, mechanical properties, energy harvesting, adaptive infrastructure

## Abstract

Metamaterials, characterized by engineered microstructures rather than chemical composition, are transforming civil infrastructure through their unique ability to achieve frequency-selective wave attenuation and programmable mechanical responses. This review provides a comprehensive overview of the applications of acoustic and mechanical metamaterials within civil engineering contexts. Acoustic metamaterials demonstrate significant potential for mitigating noise pollution in environments such as high-rise buildings, urban public areas, and transportation infrastructure by substantially enhancing sound insulation and noise reduction capabilities. Meanwhile, mechanical metamaterials, exhibiting advanced properties including shape memory, exceptional stiffness, and programmable functionality, offer novel strategies for improving structural resilience and seismic performance. Additionally, this article explores emerging opportunities in energy harvesting and adaptive infrastructure integration. Despite these advancements, critical challenges related to scalability, durability, and seamless integration with the existing infrastructure persist. Addressing these issues in future research will facilitate the advancement of sustainable, adaptive, and high-performance metamaterial solutions for modern civil infrastructure.

## 1. Introduction

Metamaterials are a new kind of functional material with special physical properties realized by artificial microstructure design. Their essential characteristics are that their unique properties come from precise microstructure design rather than inherent properties of the constituent materials [1]. According to their different physical properties, metamaterials can be divided into electromagnetic, acoustic, thermal, and mechanical metamaterials. The concept of metamaterials gained widespread attention towards the end of the 20th century, although the forms of metamaterials can be traced back to earlier times [2].

**Figure 1:**
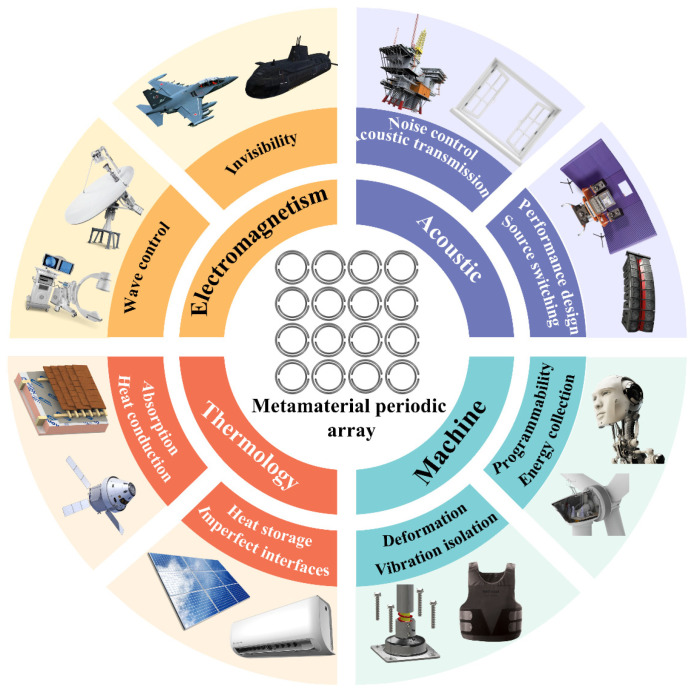
The basic structure of metamaterials and the development directions of various fields.

Figure 1 shows the development trajectory and research progress of metamaterial technology across different academic fields. The pioneering research in this field focuses on the realm of electromagnetics, particularly the innovative design of materials with negative refractive index [3]. As the theoretical framework improves, the research paradigm of metamaterials gradually extends to multi-physics fields, forming three main branches: acoustics [4], thermodynamics [5], and mechanics [6]. In the field of acoustic metamaterials, researchers have achieved precise control over sound wave propagation paths through microstructure topology optimization, breaking through the acoustic performance limits of traditional materials. For example, the design of sonic diffraction metamaterials can create nearly perfect acoustic silence zones within specific frequency bands. To realize the engineering transformation of these theoretical models, the academic community first established universal design criteria for acoustic metamaterials [7], subsequently promoting breakthroughs in applications such as noise control [8], acoustic transmission [9], acoustic performance design [10], and sound source switching [11]. The research on thermodynamic metamaterials focuses on heat flow regulation, achieving directional manipulation of heat conduction paths through heterogeneous design of materials. This type of material can not only build an efficient insulation system but also develop superconducting thermal channels, providing a brand-new solution for thermal management. The related research has been extended to the directions of thermal energy absorption [12,13], non-Fourier heat transfer [14], heat storage [15], and imperfect interfaces [16]. Additionally, inverse design research [17] has been introduced to achieve the programmable design of thermal metamaterials. In the field of mechanics, metamaterials mainly concentrate on developing materials with non-traditional mechanical responses, such as materials with negative Poisson’s ratios [18,19]. The current research mainly focuses on deformation [20,21], vibration isolation [22], energy collection [23], and programmability [24].

As advancements in material science and manufacturing technology continue to progress, research on metamaterials is moving from laboratory to practical applications. In the field of civil engineering, they show immense potential in areas such as vibration control [25], acoustic properties [26], and mechanical performance [27]. In particular, local resonance metamaterials have provided innovative solutions to break through the technical bottlenecks of traditional building materials in low-frequency vibration control and wideband noise isolation, marking a fundamental shift in the paradigm of civil engineering materials science. As shown in Table 1, the various properties of metamaterials and traditional building materials are compared. Facing the urgent demand for high-performance and sustainable engineering materials in the construction of smart cities and new infrastructure, in-depth analysis of the physical mechanism of metamaterials and the development of their engineering application technologies have become a frontier hotspot in the discipline [28]. It is worth noting that at present, systematic review studies on metamaterials in the field of civil engineering are relatively scarce. Although a large number of studies in the field of building materials have focused on the optimization of composite materials [29,30,31], their research is still limited to the performance improvement of traditional material systems and has not fully incorporated the innovative design concept of metamaterials. This study builds a knowledge bridge between theoretical innovation and engineering practice, deeply explores its engineering adaptability bottlenecks and solutions, and prospectively plans its future development path. This review is not only dedicated to improving the theoretical framework of metamaterial engineering applications but also focuses on establishing an interdisciplinary technology transformation mechanism to promote the efficient transformation of metamaterial technology into engineering practice.

## 2. Properties of Metamaterials

**Figure 2:**
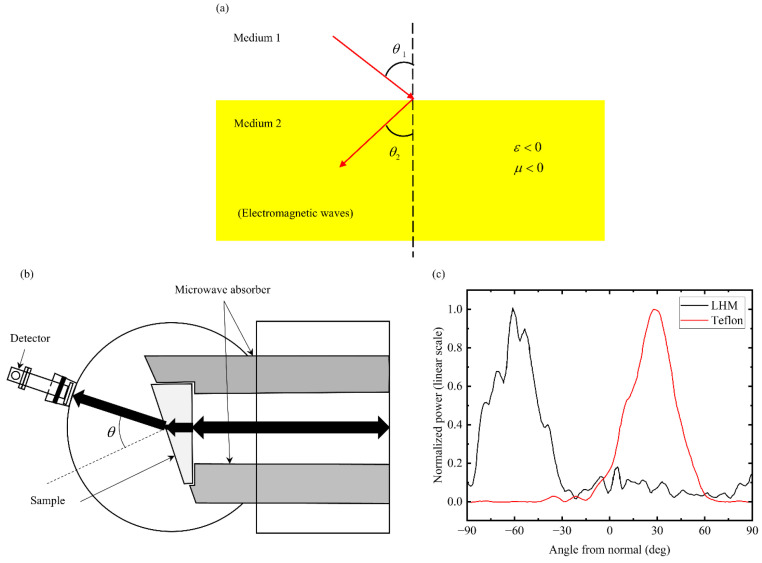
Implementation of negative refractive index in electromagnetic metamaterials: (**a**) Negative refraction phenomenon: electromagnetic waves enter the negative refraction medium and form a V-shape at the interface (θ_1_ and θ_2_ are the angles of incidence and refraction); (**b**) schematic diagram of the refraction experimental device: it consists of a wave absorber, a prism-shaped sample, a circular aluminum plate, and a microwave absorber. The black arrow represents the microwave beam [50]; (**c**) power of LHM sample (black curve) and Teflon sample (red curve) as a function of refraction angle at 10.5 GHz [50].

**Figure 3:**
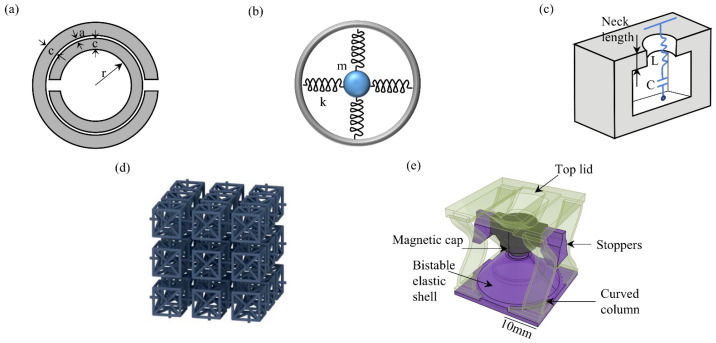
Typical metamaterial structures of different types: (**a**) copper ring resonator (electromagnetic metamaterial) [62]; (**b**) mass–spring structure (passive acoustic metamaterial) [63]; (**c**) Helmholtz resonator with inductive circuit (active acoustic metamaterial) [52]; (**d**) 3D structure cell (mechanical engineering) [22]; and (**e**) M-bit structure (mechanical engineering) [61].

The development of metamaterials originates from the realization of the negative refractive index of electromagnetic metamaterials. For electromagnetic metamaterials, dielectric constant (ε) and permeability (μ) are physical quantities that characterize the response of materials to electromagnetic waves and are constitutive parameters of the electromagnetic properties of materials [48,49]. Unlike the traditional materials that exhibit positive ε and positive μ, when both are negative, the material will show negative refractive index characteristics. The unique properties of such double-negative materials (DNG materials) or negative refractive index materials (NIM) are shown in Figure 2a. Theoretical research indicates that this abnormal electromagnetic characteristic can trigger phenomena such as perfect lens imaging and electromagnetic stealth that break through the limits of classical physics. In 2000, Shelby et al. [50] successfully observed the negative refraction phenomenon (Figure 2c) by precisely measuring the refraction angle and transmission power spectrum of electromagnetic waves in the “left-hand” material (LHM) and Teflon samples (Figure 2b). This landmark research has laid an experimental foundation for the theoretical system of metamaterials. Its physical mechanism can be traced back to the design of sub-wavelength resonant units. When the concave copper ring structure (Figure 3a) induces an induced current in a specific frequency band, the phase propagation direction of the electromagnetic wave shows an opposite characteristic to the energy propagation direction. At this time, the equivalent size of the copper ring structure significantly exceeds its physical scale, triggering an abnormal electromagnetic response where ε and μ are both negative.

In view of the mathematical isomorphism of sound waves and electromagnetic waves at the level of the wave equation, the two have a profound physical correspondence in the mechanism of propagation regulation. Through the multi-dimensional synergy of theoretical derivation, experimental verification, and engineering practice, the establishment of a new acoustic wave regulation system has emerged. Acoustic metamaterials achieve precise control of elastic wave propagation through subwavelength structure design, and their physical nature is regulated by Newtonian dynamics equations, fluid continuity equations, and equations describing thermodynamic states. Distinct from their electromagnetic counterparts, acoustic metamaterials are characterized by two fundamental parameters: mass density (ρ) and bulk elastic modulus (K). These parameters not only define the basic acoustic response characteristics of the material but also provide a theoretical basis for constructing anomalous acoustic phenomena. The specific expressions for ρ and K can be expressed by Equations (1) and (2).

(1) $$\rho =\frac{m}{V}$$

(2) $$K=-V\frac{dP}{dV}$$

where m is the mass, V is the volume, and P is the pressure.

However, the design of acoustic metamaterials often relies on complex geometries and complex material composites. Consequently, the effective mass density $\rho_{eff}$ and $K_{eff}$ can be calculated through a volumetric weighted average calculation approach, which is expressed by Equations (3) and (4).

(3) $$\rho_{eff}=\sum_{i}\phi_{i}\rho_{i}$$

(4) $$K_{eff}=\sum_{i}\phi_{i}K_{i}$$

where $\phi_{i}$, $\rho_{i}$, and $K_{i}$ are the volume fraction, mass density, and elastic modulus of material i.

The physical mechanism by which acoustic metamaterials achieve negative constitutive parameters stems from the design of their subwavelength resonant structure. By introducing artificial microstructures, such as membranes [51] and Helmholtz resonators [52], the parameter limitations of traditional materials can be broken through. The mass–spring resonant system shown in Figure 3b is composed of a mass sphere (m) and a spring with an elastic coefficient of k. When the forced vibration phase of the system lags behind the excitation, its inertial response shows an inverse phase characteristic, thereby generating a negative equivalent mass density in a specific frequency band. Figure 3c illustrates a Helmholtz resonator, which is composed of a cavity and an inductor–capacitor (LC) circuit. Under the excitation of sound pressure, the gas inside the cavity flows, resulting in compressive deformation. When the characteristic size of the resonator is much smaller than the working wavelength, this subwavelength structure behaves as a uniform medium macroscopically, and its strong dispersion characteristic causes the system to present a negative elastic modulus response near the resonant frequency.

This local resonance mechanism can be quantitatively described. When the unit cell size meets one-tenth of the wavelength, resonance excitation occurs, causing oscillation and endowing the material with effective parameters. The mass density $\rho_{effs}$ and bulk modulus $K_{effs}$ can be expressed by Equations (5) and (6) [53].

(5) $$\rho_{effs}=\rho_{0}\left[1-\frac{F_{t}\omega_{1}^{2}}{\omega^{2}-\omega_{1}^{2}+iT_{t}\omega }\right]$$

(6) $$\frac{1}{E_{effs}}=\frac{1}{E_{0}}\left[1-\frac{F_{p}\omega_{1}^{2}}{\omega^{2}-\omega_{1}^{2}+iT_{p}\omega }\right]$$

where $\rho_{0}$ and $E_{0}$ are the initial mass density and elastic modulus, respectively, $\omega_{1}$ is the resonant angular frequency, ω is the operating frequency, $F_{t}$ and $F_{p}$ are the geometric factors, $T_{t}$ and $T_{p}$ are the dissipation loss, and i is the imaginary quantity.

Consequently, acoustic metamaterials exhibit properties such as negative refractive index, anomalous Doppler effect, amplified evanescent waves, and perfect sound absorption [54,55].The negative refractive index is the backpropagation of the sound wave, and according to the wave equation of the sound wave, the refractive index can be expressed by Equation (7).

(7) $$n=\sqrt{\frac{K}{\rho }}$$

where n, K, and ρ are the refractive index, elastic modulus, and mass density.

The anomalous Doppler effect indicates that when the observer approaches the sound source, it shows a reverse decrease in the received frequency, while when moving away, the frequency increases instead. The physical essence of this counterintuitive phenomenon stems from the reverse regulation mechanism of the metamaterial on the direction of the sound wave vector. More importantly, evanescent waves attenuate exponentially at the medium interface and are difficult to detect effectively in traditional material systems due to the limitations of the diffraction limit. By constructing the acoustic impedance gradient matching structure, acoustic metamaterials can convert evanescent waves into propagating waves, increasing their transmission distance by more than two orders of magnitude.

**Figure 4:**
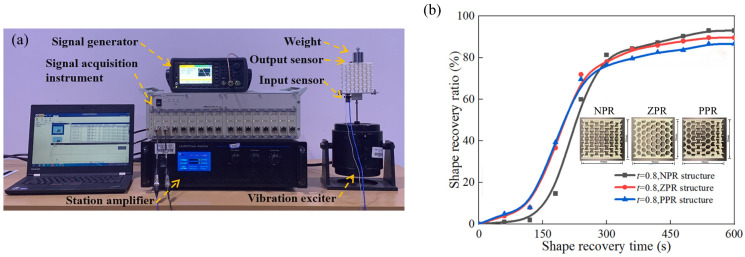
Evaluation of structural deformation with different Poisson ratios: (**a**) stress–strain test equipment; (**b**) curve of the relation between deformation recovery degree and recovery time of three honeycomb metamaterials (t is for thickness) [36].

The innovative breakthroughs in mechanical metamaterials stem from a profound understanding of the biomimetic multi-scale structures of natural biomaterials. Unlike traditional materials that rely on composition to regulate mechanical properties, biological systems represented by honeycomb materials have evolved over hundreds of millions of years to form cross-scale topological optimization structures [56]. This structure–function integrated design concept provides important inspiration for artificial metamaterials. With the breakthroughs in nanoscale additive manufacturing technology, especially multi-material 3D printing [57], researchers have been able to precisely construct multi-level cell structures with periodic/quasi-periodic arrangement characteristics (Figure 3d), achieving mechanical response properties that traditional materials cannot reach. Through the strategic design of cell topological configuration, mechanical metamaterials can break through the framework of classical continuum mechanics and exhibit anomalous mechanical behaviors, such as negative Poisson’s ratio (NPR), negative compressibility, adjustable stiffness, and zero shear modulus. When NPR material is axially stretched, it expands laterally rather than contracts. This “pull-expansion effect” stems from the synergistic deformation mechanism of special cell structures such as concave hexagons. Negative compressible materials expand in volume under pressure, and the physical essence lies in the cell reconstruction process induced by buckling. As shown in Figure 4, the comparative study indicates that NPR metamaterials exhibit superior shape recovery capabilities compared to the zero Poisson’s ratio (ZPR) and positive Poisson’s ratio (PPR) systems. These special properties stem from the size effect; that is, when the characteristic size of the material enters the nanometer scale, the surface energy-dominated mechanism leads to a size-dependent strengthening phenomenon in its mechanical properties. Deformation can either strengthen [58] or weaken [59], and stiffness can markedly increase [60]. This is due to material defects, like cracks and voids, which reduce actual strength below theoretical values. However, reducing size can mitigate these defects, significantly enhancing strength. For metamaterials, the size effect becomes pronounced at the characteristic length scale. Chen et al. [61] verified this principle through the design of a magnetic coupling bistable structure. The microstructure unit composed of an elastic shell, a magnetic top cover, and a curved pillar (Figure 3e) exhibited an unconventional stiffness-strengthening effect after periodic assembly. This cross-scale design strategy provides theoretical guidance for the development of a new generation of high-strength and lightweight engineering materials.

The design paradigm of metamaterials essentially breaks through the research and development framework of traditional engineering materials. Its innovation is reflected in the paradigm shift composed of four dimensions: design philosophy, material system, functional realization path, and engineering adaptability. Table 2 shows the specific differences between metamaterials and traditional engineering material design strategies.

**Table 2:** Comparison of design strategies for conventional materials and metamaterials.

Dimensionality	Traditional	Metamaterial
Design objective	Static strength, stability, economy	Dynamic response [64], multifunctional integration [65], extreme environmental adaptability [38]
Freedom to innovate	Limited by inherent properties of materials	Defining new properties from microstructure and material combinations
Environmental interaction	Passively endure	Active sensing and adaptation [38]
Material system	Single homogeneous material or simple composite	Heterogeneous composite of multi-materials [66]
Upper limit of performance	Limited by material intrinsic properties	Defined by structural design
Environmental adaptability	Dependence on external protection	Intrinsic adaptation [67]
Function expandability	Low (external equipment required)	High (structure intrinsic multifunction)
Energy dependence	High (external power required)	Low (self-powered potential)

## 3. Metamaterials in Vibration Control

Metamaterial technology is driving the innovation of building seismic engineering, enhancing the safety threshold and service performance of structural systems under complex dynamic loads, such as strong earthquakes, hurricanes, and traffic pulsations. Under this technical framework, acoustic metamaterials and mechanical metamaterials constitute two core branches. The former supposes the propagation of elastic waves by constructing subwavelength phonon crystal bandgaps, while the latter realizes the directional conversion of vibration energy with the help of multi-level energy dissipation structures. The two types of materials show significant complementary characteristics in the performance dimension. Although acoustic metamaterials have a higher single-frequency attenuation intensity, their bandgap width is usually limited to 10–15% of the fundamental frequency. However, mechanical metamaterials can cover a frequency range of 2 to 3 orders of magnitude through a wideband dissipation mechanism, but their peak attenuation efficiency is relatively low. Therefore, acoustic metamaterials are suitable for lightweight building components that are sensitive to low-frequency vibrations, such as large-span spatial structures and curtain wall systems, while mechanical metamaterials are more appropriate for load-bearing structural systems that need to withstand wide-frequency vibrations, such as the core tubes of high-rise buildings and bridge bearings. While mechanical metamaterial vibration isolation will be discussed in the context of mechanical properties, this section focuses primarily on acoustic metamaterial vibration control.

The vibration control of acoustic metamaterials relies on the physical effect of local resonance (LR). Negative equivalent elastic modulus is induced through the design of subwavelength resonant units, forming a band gap for elastic wave propagation within a specific frequency band. In engineering practice, the topological configuration of the resonant unit directly affects the system performance. Although the embedded resonator has the advantage of compact space (volume proportion < 5%), there is a risk of structural integrity damage and limited accessibility for operation and maintenance. External resonators ensure structural protection performance through modular design and significantly enhance maintenance convenience, but they require an additional 10% to 15% of building space. This design choice needs to be based on the life cycle cost model and achieve a dynamic balance among space utilization efficiency, structural reliability, and operation and maintenance economy.

### 3.1. Vibration Control Using Cavity Resonators

**Figure 5:**
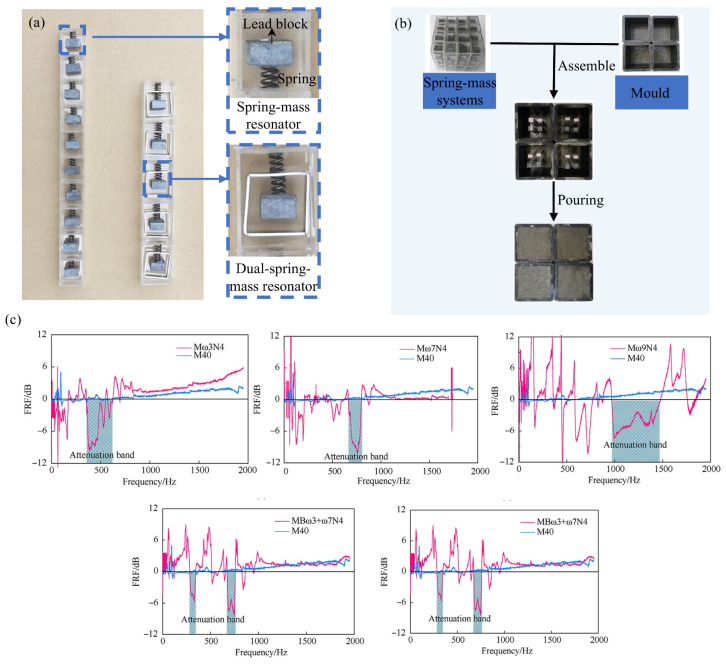
Meta-concrete application: (**a**) schematic diagram of the spring–mass resonator; (**b**) meta-concrete production process; (**c**) damping effect test (M40 is an ordinary mortar with a pressure strength of 40 Mpa; Mω3N4, Mω7N4, and Mω9N4 are supermortar blocks composed of four resonators with a resonant frequency of 305 Hz, 707 Hz, and 903 Hz, respectively) [68].

The technological innovation of acoustic metamaterials in civil engineering focuses on the breakthrough research of resonant unit configuration design and bandgap regulation mechanism. The current mainstream design systems of cavity resonators include five major types of topological configurations: closed spring–mass systems [68], cell beam systems [69], cell truss systems [70], end-loaded cantilever resonator systems [71], and cell rod systems [72]. Among them, the closed spring mass resonator has gained key applications in the field of concrete-based metamaterials due to its compact structure. Liu et al. [68] successfully developed metamaterial concrete with a frequency-selected vibration damping function by innovatively constructing a periodic composite structure of the spring–mass resonant system and the concrete matrix. Vibration experiments demonstrated that the inclusion of resonators significantly attenuates stress waves at specific frequencies, offering valuable insights for earthquake-resistant construction, but the static compressive strength is affected by the relevant design of the structure (Figure 5). This reveals the core contradiction of the integrated design of structure and function. To break through this bottleneck, Mitchel et al. [73] proposed a gravel substitution strategy, which achieves wideband vibration suppression while maintaining the material’s load-bearing capacity by randomly distributing micro-resonators in the concrete matrix. Numerical simulations show that this type of metamaterial concrete can reduce the transferred energy, and its dissipation mechanism stems from the nonlinear interfacial slip effect between the resonant unit and the matrix. The performance of metamaterials depends on their microstructure, and 3D printing is the key technology for their practical application. Quinteros et al. [74] utilized 3D printing and structural topology to develop a honeycomb truss core, resulting in a lightweight composite material with vibration absorption properties while maximizing the band gap, offering a novel approach for seismic design in civil engineering. Bandgap regulation technology is promoting seismic metamaterials to enter the stage of multi-band coordinated protection. The development of V/N gradient metamaterials by Su et al. [75] marked an important breakthrough in this field. The V-shaped configuration generates a directional band gap with attenuation in a specific frequency band, while the effective bandwidth can be increased by introducing the N-shaped topology extension. More notably, by adopting the concrete-based gradient array design, a multi-bandgap coupling effect is achieved on the premise of minimizing the engineering cost to the greatest extent, thereby enhancing the overall vibration suppression efficiency. However, most of the existing studies are limited to the category of linear response and show significant performance attenuation when dealing with strong nonlinear ground motion. Frontier research indicates that the introduction of a nonlinear stiffness regulation mechanism can construct an amplitude–frequency adaptive metamaterial system, whose energy consumption density can be significantly increased compared with linear systems. This points out the development direction for breaking through the existing technical bottlenecks.

### 3.2. Vibration Control Using External Cavity Resonators

**Figure 6:**
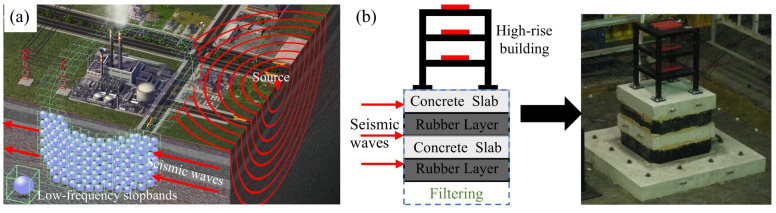
Different structural vibration isolation schemes: (**a**) earthquake stealth [87]; (**b**) periodic structure (the left picture is a physical picture, and the right picture is a schematic) [88].

**Figure 7:**
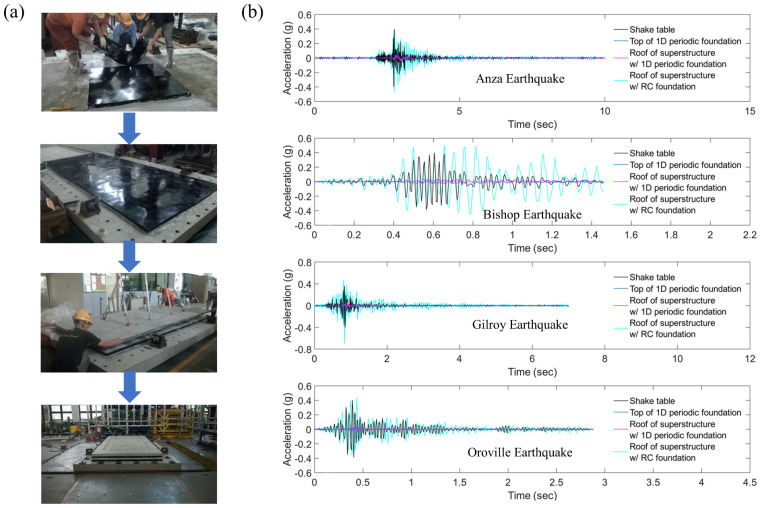
Periodic foundation isolation seismic wave verification: (**a**) periodic base assembly process (gray is the concrete layer, black is the polyurethane layer); (**b**) simulated seismic experiment results (1D is one-dimensional, RC is reinforced concrete) [89].

External cavity resonators are categorized into meta-truss [76], meta-sandwich [77], meta-beam [78], and seismic metamaterials [79]. Among them, seismic metamaterials dominate due to their technical maturity and wideband performance in the field of large-scale building isolation. Its application in seismic resistance of civil engineering mainly lies in the seismic stealth and isolation of periodic structures. For existing buildings, the low-frequency stopband technology based on a three-dimensional resonator array (Figure 6a) achieves foundation stealth in seismic waves through underground implantable design. Colombi et al. [80] coupled the forest root network with resonant resistance bands in a design, which not only expanded the band gap width but also achieved the collaborative resonance between artificial structures and natural ecosystems, providing a new paradigm for green seismic engineering. For the more destructive Rayleigh surface waves, the academic community has broken through the technical bottleneck through multi-physics field coupling design [81]. Zeng et al. [82] revealed the quantitative relationship between the height of the resonant element and the center frequency of the band gap through a parametric model. Lou et al. [83] developed a stratified foundation nonlinear metamaterial system, adopted gradient viscoelastic interlayers to enhance the Rayleigh wave attenuation efficiency, and established a mapping database of soil modulus—band gap characteristics through finite element inversion. For the seismic resistance of new buildings, the periodic structure isolation system achieves extraordinary isolation performance through the design of the fundamental frequency band gap [84]. The concrete–rubber composite periodic foundation shown in Figure 6b was verified by the shaking table experiment (Figure 7a), and under the excitation of seismic waves, the attenuation rate of the peak acceleration of the structure reached 88.12–91.55% (Figure 7b). Numerical simulations further indicate that this system can reduce the displacement response of high-rise buildings [85], and by presetting the microcrack topology, it can induce secondary scattering of stress waves and increase the isolation bandwidth [86]. These findings provide crucial technical support for designing high-rise building foundations in seismic zones. However, the current concrete–rubber system has significant technical bottlenecks. The cumulative plastic deformation under cyclic loading leads to the attenuation of isolation performance with the service cycle, and the total life cost is higher than that of traditional foundations. The breakthrough direction lies in the development of self-healing material systems, such as shape memory alloys–piezoelectric ceramic composite resonators, which can achieve performance regeneration through phase change energy dissipation and crack self-repair.

In the field of seismic resistance of superstructures, periodic structures, with their modular design characteristics, are becoming the core technical path of vibration reduction systems. In terms of technological breakthroughs, Yu et al. [90] integrated Bragg dispersion and LR mechanisms to address pipeline bending vibrations in periodic pipeline systems. In response to the special working conditions of marine engineering, Asiri and AL-Zahrani et al. [91] designed periodic support columns for offshore structures to dampen wave-induced vibrations. Huang et al. [92] developed a composite periodic steel column, which represents another important direction. By filling the hollow section with gradient sound-absorbing foam and installing an internal steel resonant ring, it reduces the low-frequency vibration response while maintaining the axial bearing capacity. This structure–function integrated design can precisely match the characteristics of the ground motion spectrum by adjusting the wall thickness and filling rate of the unit, providing a new paradigm solution for the customized protection of major infrastructure [85]. Although the above achievements have verified the engineering feasibility of the periodic structure, the current research lacks the co-design criterion of load-bearing and vibration damping, and the construction feasibility assessment system is not perfect. To solve these problems, it is necessary to establish a multi-objective optimization model and explore new composite material systems to inject new impetus into the seismic design of the superstructure.

## 4. Metamaterials in Acoustic Performance

Noise pollution, as an interdisciplinary technical problem, has long plagued the fields of architectural acoustics, environmental engineering, and urban infrastructure [93]. Traditional noise reduction technologies rely on porous sound-absorbing materials and cavity resonance structures, and their low-frequency noise reduction efficiency is limited by the physical limit of the law of mass. The emergence of acoustic metamaterials has broken through this predicament and achieved the regulation of the sound wave propagation path. Various structures have been developed to achieve the desired acoustic properties, including Helmholtz resonators [94], half-wavelength and quarter-wavelength resonators [95], rolled resonators [96], local resonance resonators [97], and phonon crystals [55].

Helmholtz resonators achieve the absorption of sound waves at specific frequencies through the neck cavity structure. Half/quarter wavelength resonators, based on the standing wave principle, play a significant role in civil engineering and are widely used in building curtain walls and piping systems. Rolling resonators achieve acoustic wave phase delay compensation with the aid of 3D-printed surface topology, and their surface density is lower than that of traditional structures. Local resonant metamaterials generate low-frequency band gaps through subwavelength unit design, while phonon crystals control high-frequency noise by using the Bragg scattering mechanism. The engineering adaptability of these technologies shows significant differences. The local resonance system dominates in the field of traffic noise barriers, and its isotropic characteristics are adapted to the linear structure of the road. Phonon crystals, with their anisotropic regulation ability, are widely used in the suppression of high-frequency noise in industrial workshops. This section is discussed based on different application parts.

### 4.1. High-Rise Building Application

**Figure 8:**
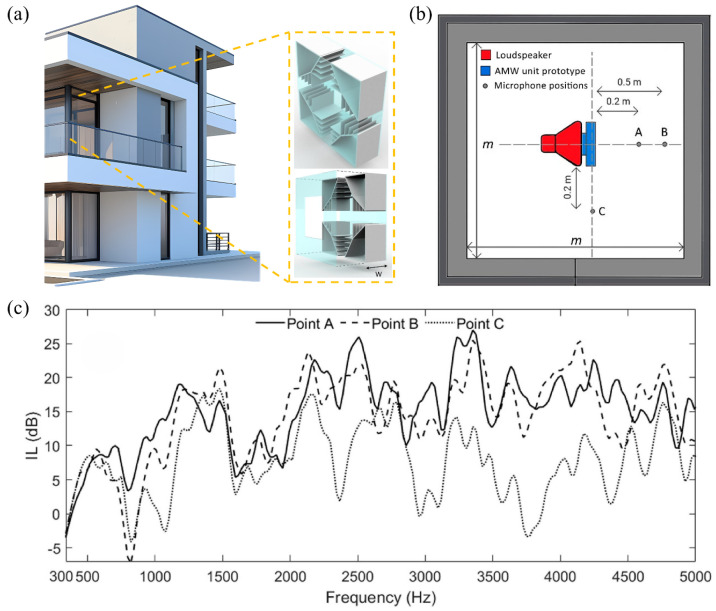
Meta-window design and acoustic testing: (**a**) meta-window model schematic (W is the sound wave width, W = 0.19 m); (**b**) sound insulation experiment design scheme (m = 2 m, the black box is the silent room); (**c**) test insertion loss (IL) at positions A, B, and C to show acoustic performance [26].

**Figure 9:**
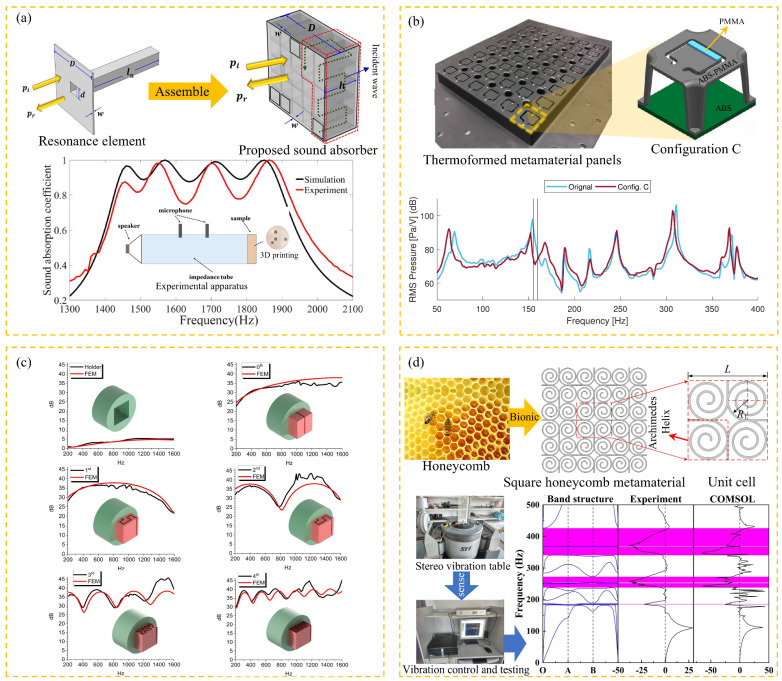
Acoustical metamaterials and experimental results for architectural compliance design: (**a**) Space design of sound absorber (*p_i_* represents the incident sound wave, and *p_r_* represents the reflected sound wave. w and h represent the width and thickness of the cell of the sound absorber, respectively, D represents the thickness of the sound absorber, and the green dashed line indicates the extended propagation distance of the sound wave. d is the inner diameter of the tubule, and l_n_ is the length of the tubule) [99]. (**b**) Thermoformed panel cell (ABS-PMMA is Acrylonitrile Butadiene Styrene–Polymethyl Methacrylate) [104]. (**c**) Fractal metamaterials (black is the transmission loss experimental results, red is the finite analysis results) [101]. (**d**) Honeycomb acoustic metamaterials (L is the cell length, and R1 is the helix radius) [103].

**Figure 10:**
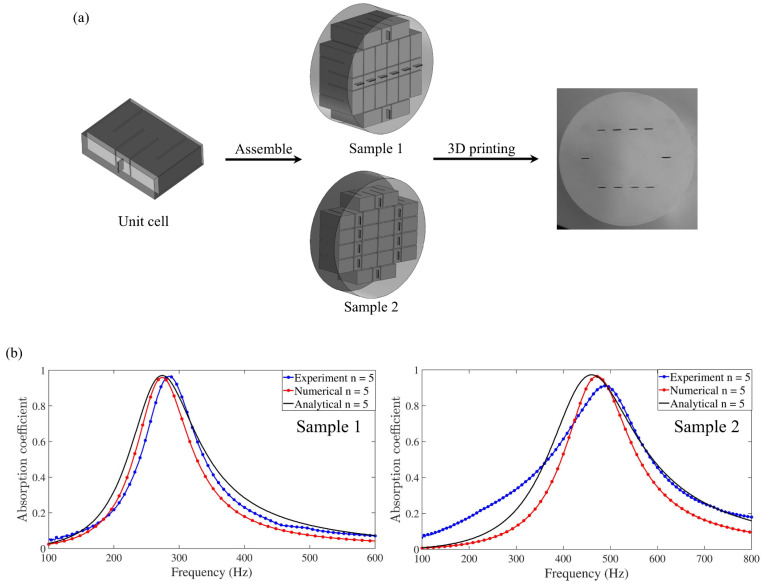
Structural design and sound insulation test of rolling resonators: (**a**) manufacturing process of metamaterial with labyrinth rolling resonator (sample 1 consists of 8 unit cells, and sample 2 consists of 10 unit cells); (**b**) theoretical, numerical, and experimental behavior of sound absorption coefficient of labyrinth metamaterials [100].

In the field of urban architectural acoustics engineering, the innovative application of acoustic metamaterials is reconstructing the noise protection systems of high-rise buildings and apartments [98]. As shown in Figure 8, the acoustic meta-window (AMW) technology breaks through the physical limit of traditional soundproof windows and achieves noise suppression in a wide frequency range of 300–5000 Hz while maintaining the ventilation rate. This kind of integrated acoustic design requires interdisciplinary collaboration among architects, structural engineers, and acoustic experts to achieve the coupling optimization of multiple physical fields such as sound, force, and heat. For non-load-bearing walls, Gao et al. [99] proposed a labyrinth metamaterial structure. This design transforms the traditional cavity into a broadband sound absorber through a three-dimensional spiral channel topology, achieving sound absorption in the 1.4–1.9 kHz frequency band and enhancing the structural stiffness (Figure 9a). Compared with traditional sound-absorbing walls, this system uses less material and has a lower carbon footprint, demonstrating the potential of green building acoustic solutions. The core challenge of noise control in high-rise buildings lies in the efficient suppression of low-frequency noise (50–500 Hz). In 2021, Almeida et al. [100] designed a micro-perforated plate–rolling resonator composite structure (Figure 10), providing an innovative path for this. This system integrates the Helmholtz resonance mechanism of the micro-perforated plate and the phase modulation characteristics of the rolling resonator. The noise reduction effectiveness in the 100–600 Hz frequency band has been verified through impedance tube experiments. It laid the foundation for the building skin system.

The structural integration technology of acoustic metamaterials is reshaping the acoustic design of building envelope systems. As shown in Figure 9b, the thermoformed metamaterial panel breaks through the limitations of the traditional rubber-based sound insulation structure. Through the design of a periodic micro-protrusion array, it achieves a reduction in surface density while reducing the peak value of the first mode of structural vibration by 8 dB [101,102]. This metal-based metamaterial panel is manufactured by a hot stamping process, reducing production costs and laying the foundation for industrial-scale application. The introduction of fractal geometry has opened up a new dimension for the design of acoustic metamaterials. Comandini et al. [101] utilized 3D printing to characterize fractal patterns on periodic metal bases, experimentally comparing orders from zero to four. The fractal order significantly influences the number of transmission loss peaks (Figure 9c). This multi-scale fractal topology achieves wideband regulation capabilities that are difficult for traditional structures to reach by stimulating multi-level local resonances. For lightweight applications, bionic-inspired honeycomb and spiral structures provide a combination of light weight and local resonance [103]. The Archimedes helical honeycomb structure shown in Figure 9d achieves an improvement in the stiffness–mass ratio through topological optimization, and its nested helical units form multiple low-frequency bandgaps in the low-frequency band of 200–500 Hz. This structure is well-suited for lightweight construction and railway applications. However, current research mostly focuses on single-objective optimization and lacks a multi-constraint collaborative design model of stiffness–sound insulation–lightweight, which limits its application in building integration. Breakthrough progress may come from the innovative application of new composite materials. Graphene-reinforced metamaterials can increase surface stiffness while reducing density, while basalt fiber-braided meta-surfaces have both weather resistance and restorability.

### 4.2. Public Place Application

**Figure 11:**
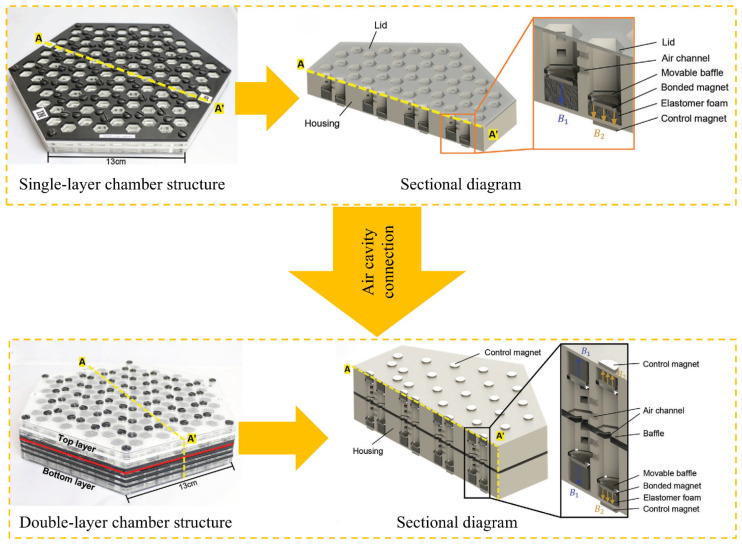
Schematic diagram and sectional diagram of acoustic chamber structure composed of magnetic active acoustic topological transistors [105].

**Figure 12:**
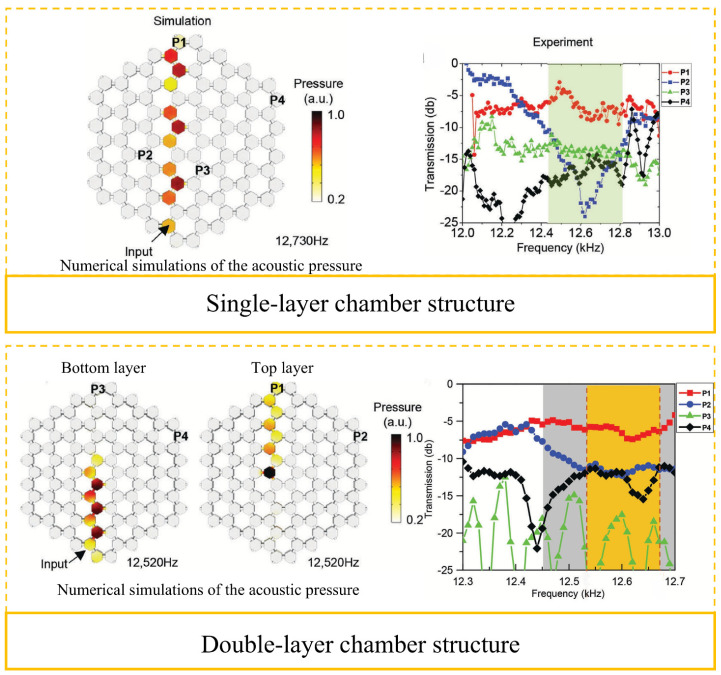
Sound pressure simulation of single and double chamber magnetically excited gas chambers and experimental results of acoustic permeability at P1, P2, P3, and P4 frequencies [105].

The revolutionary application of acoustic metamaterials in public building acoustic engineering is breaking through the theoretical boundaries of classical wave regulation. Lee et al. [105] developed magnetic active acoustic topological transistors, which induced the quantum Valley Hall effect through magnetic field regulation, achieving topological phase transition regulation of the acoustic wave transmission path. Despite its high manufacturing cost, its unique sound field space programming capability makes it irreplaceable in high-end cultural buildings, such as opera houses and concert halls. As shown in Figure 11, the structure is composed of an array of Helmholtz acrylic resonant cavities, each of which is equipped with a magnetostrictive volume regulator. The double-layer topology design realizes the conversion of acoustic wave modes through sub-wavelength air ducts, enabling effective regulation of the bandwidth to expand the fundamental frequency. The simulation of the sound pressure field and the permeability experiment (Figure 12) show that the P1 cavity exhibits selective transmission characteristics at a specific resonant frequency. By optimizing the cavity density, this system can increase the acoustic frequency range and provide a new solution for customizing the acoustic environment of large public spaces. The current technical bottleneck lies in the energy consumption of the active regulation system. The piezoelectric fiber and friction nanogenerator are integrated to realize self-powering by acoustic–mechano–electric multi-physical field coupling. This energy-autonomous acoustic metamaterial system will enhance the sustainability of architectural acoustics.

### 4.3. Road Traffic Application

**Figure 13:**
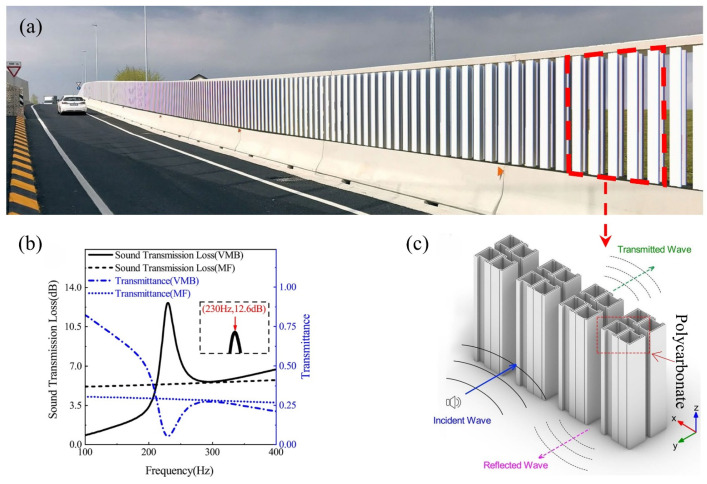
Structural design of metamaterial barrier and experimental results of sound insulation: (**a**) ventilated metamaterial barrier (VMB) for traffic noise; (**b**) sound transmission loss and transmittance curves of the VMB and the MF; (**c**) periodic bilateral cell structure arranged infinitely along the x direction [46].

Acoustic metamaterials have demonstrated revolutionary engineering value in the field of traffic noise control, and their innovative design breaks through the binary opposition predicament of “noise reduction–ventilation” of traditional sound barriers. As shown in Figure 13, the ventilated acoustic metamaterial barrier adopts polycarbonate wavelength resonance units. Through the Helmholtz resonance and micro-perforated plate coupling mechanism, the precise suppression of low-frequency traffic noise ranging from 100 to 400 Hz is achieved while maintaining the airflow permeability rate. The measured engineering data show that the insertion loss of this system at the characteristic frequency of 230 Hz reaches 13 dB, which is 40% higher than that of the traditional melamine foam (MF) barrier. This barrier has significantly improved the acoustic environment quality of the residential areas along the line.

The optimal design of sound barriers requires a systematic consideration of key physical factors, such as multiple scattering resonance coupling, cavity topological parameterization, and incident wave vectors [106]. Aiming at the frequency domain characteristics of railway noise, Zhang et al. [107] introduced the genetic algorithm neural network into the reverse design of metamaterials. Through multi-objective optimization, a non-intuitive topological configuration was obtained, and its effective band gap was broadened to 1295 Hz. He et al. [108] proposed an ecological acoustic metamaterial system and constructed an optimization model including ecological acoustic parameters such as tree density, canopy volume, and planting spacing, providing a theoretical framework for the acoustic design of green infrastructure. Although acoustic metamaterials demonstrate significant technical advantages, their engineering adaptability still faces multi-dimensional challenges, such as cost, durability, and environmental adaptability. The breakthrough lies in the combination of interdisciplinary technologies and the integration of adaptive systems. In addition, bionics can become a new idea for the design of metamaterials, and other fields can solve key technical problems through bionic technology.

### 4.4. Tunnel Engineering Application

**Figure 14:**
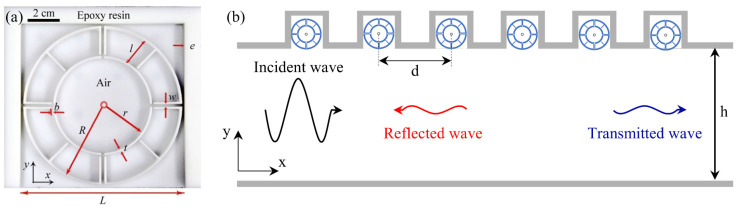
Schematic diagram of acoustic metamaterial structure in tunnel: (**a**) mie resonance cell physical diagram; (**b**) tunnel diagram with mie resonance cells [109].

**Figure 15:**
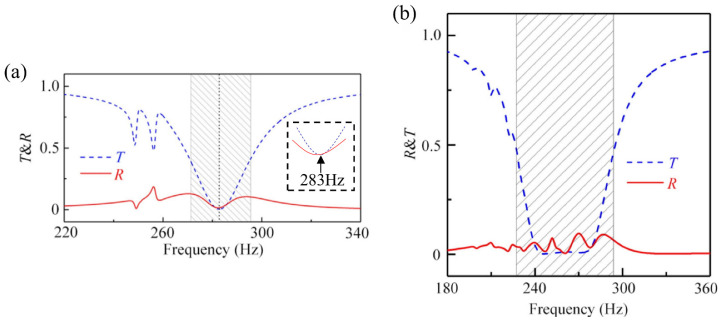
Tunnel transmittance (T) and reflectance (R) spectra for different cell numbers: (**a**) two-cell simulation; (**b**) six-cell simulation [109].

In tunnel engineering, metamaterials can be integrated into tunnel walls to diminish internal sound reflection and reverberation, thereby reducing noise levels at tunnel exits [109]. As shown in Figure 14, the annular gradient multi-cavity resonant unit is precisely manufactured by 3D printing with epoxy resin. These cells have openings of length L on the bottom side. The multi-cavity structure is separated by four channels whose thickness is w, and opening width and radial length are b and l, respectively. The overall frame thickness is t, and the medium in the cavity is air. Simulations of the cells’ transmittance and reflectivity within a tunnel environment indicate that two cells achieve optimal sound absorption at 284 Hz (Figure 15a). With six cells, the sound absorption coefficient within the 240 Hz to 300 Hz range can reach 0.9. Increasing the number of cells not only broadens the band gap but also allows for customization based on the frequency range required for specific engineering applications (Figure 15b). Tunnel application research is still at a limited experimental level, but considering the actual environment of the tunnel, the use of self-healing materials to extend the service life and the development of compact and modular designs that can be flexibly installed in a limited space hold significant potential.

### 4.5. Other Engineering Applications

**Figure 16:**
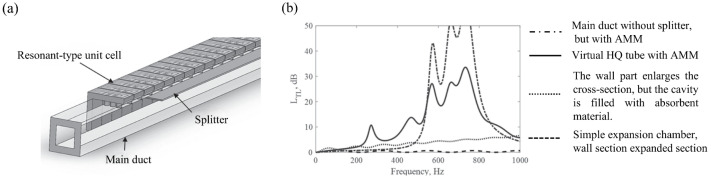
Structure of Herschel–Quincke tube and experimental results of sound insulation: (**a**) aerial view (the unit cell consists of quarter-wave tube resonators with three different lengths); (**b**) acoustic transmission loss (TL) of different silencers [112].

Operations such as mechanical vibrations and fluid movements in pipelines generate noise that impacts work, residential, and ecological environments. Regarding the aerodynamic noise of the blade system, Liu et al. [110] simulated the acoustic excitation response of moth wings to optimize thickness variation and local resonance; this has been successfully applied to the blade optimization design of pump and valve systems. In pipeline systems, Herschel–Quincke (HQ) tube walls fitted with acoustic metamaterial (AMM) cells can reduce fluid noise by 5 to 30 dB within a specific frequency range (Figure 16). Compared to other designs, this structure only increases the original pipe volume by 40%, making it suitable for industrial equipment. Papathanasiou et al. [111] confirmed through the analysis of periodic hierarchical branch profiles that HQ tubes can improve the turbulence noise attenuation efficiency of high-pressure hydraulic systems. Cost and productivity are key factors in industrial applications. At present, the resonator has been miniaturized while ensuring noise reduction, but it is limited to sample experiments, and new materials, such as self-healing materials and superconducting materials, should be introduced in the future to improve structural energy consumption and enhance high temperature resistance and corrosion resistance. In addition, in the future, it can be effectively integrated with industrial equipment to reduce energy consumption and build an integrated noise control system.

## 5. Metamaterials in Mechanical Responses

With the diversified demands of architecture, material design focuses on multi-objective optimization to achieve the unity of mechanics, function, and environmental benefits. Traditional building materials have single characteristics and poor sustainability. Mechanical metamaterials have broken through the performance threshold of the materials themselves through the structural size effect. Their application in this domain is primarily driven by their unique deformation properties, such as shape memory, exceptional stiffness and toughness, and distinctive mechanical responses. This section will discuss these three different characteristics.

### 5.1. Shape Memory

**Figure 17:**
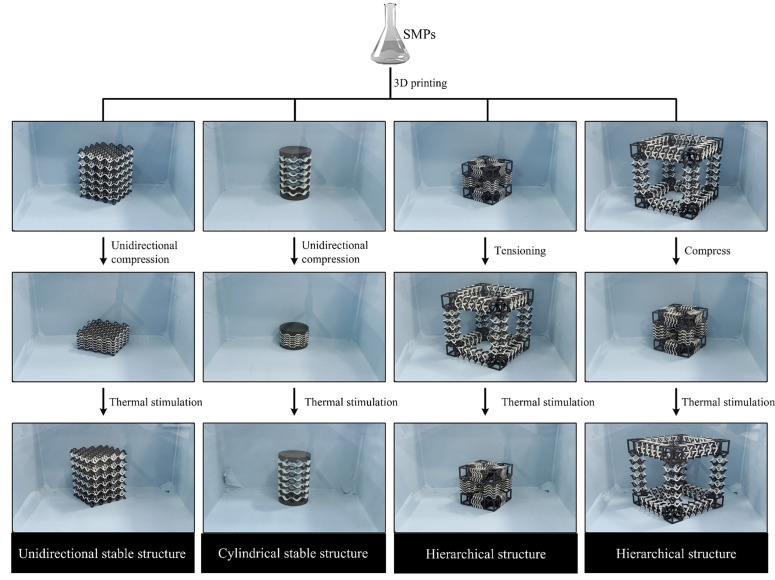
Three-dimensional mechanical metamaterial fabrication of different shapes and demonstration of shape memory properties [120].

**Figure 18:**
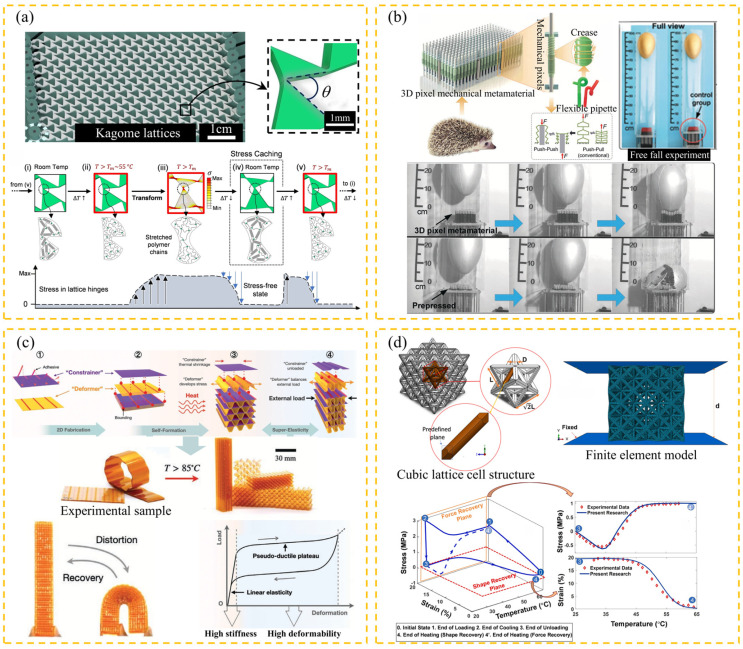
Design and properties of SMP-based metamaterials: (**a**) mechanical metamaterials in the form of kagome lattices (*θ* is the triangular element angle, *T* is the lattice temperature, *T_m_* is the SMP melting temperature, ∆*T* is the temperature change value, and σ is the stress intensity) [37]; (**b**) 3D pixel mechanical metamaterials (*F* is the force) [113]; (**c**) self-forming lightweight mechanical metamaterials (*T* is room temperature) [33]; (**d**) eight-element truss structure (*L* is the pillar length, *D* is the pillar diameter, and *d* is the lattice length) [114].

The essence of the shape memory effect (SME) is that materials achieve reversible regulation of “deformation–storage–recovery” through metastable phase transformation mechanisms. Its engineering realization relies on three major composite materials: shape memory polymers (SMPs), shape memory alloys (SMAs), and shape memory ceramics (SMCs). Among them, SMPs have demonstrated unique advantages in civil structures due to their high shape recovery rate, low density, and cost-effectiveness. As shown in Figure 17, the SMP mechanical metamaterial based on 3D printing has restored its current state after loading, breaking through the deformation limit of traditional materials. For example, Jolly et al. [37] designed a kagome lattice based on SMPs that can accommodate self-stress states and shape adaptation (Figure 18a). Pan et al. [113] developed mechanical pixel metamaterials to enhance the shear stress strength. The golden egg impact test indicated that it had good buffering performance and was suitable for the core layer of building isolation bearings (Figure 18b). Wu et al. [33] created a compressed shrink film into a volume structure with self-adaptive stiffness after loading, providing reliable solutions for lightweight and shape memory panel designs in engineering (Figure 18c). Pirhaji et al. [114] studied the shape memory characteristics of different shapes and high-stress compression using SMPs’ eight-element truss structure, achieving high shape recovery (Figure 18d). This can be applied to bar design and other tensioning characteristics in civil engineering.

Local deformation of structures often leads to overall system instability. Bauer et al. [115] proposed tension-regulating metamaterials. Through the topological optimization design of the internal prestressed network, this material system significantly enhances the damage tolerance of building structures, effectively suppressing the crack propagation and buckling instability modes of wall and column structures, thereby ensuring the safety of structural service. With the deep integration of active materials and additive manufacturing technology, 4D printing technology emerged [116]. By embedding stimulation-responsive material components in the three-dimensional printing process, it achieves the spatiotemporal controllability of structural dynamic reconstruction ability and adaptive function. Xu et al. [36] further constructed metamaterials with gradient Poisson’s ratio characteristics by using 4D printing technology. Through finite element numerical simulation, they systematically studied their multi-level deformation mechanism, shape memory recovery rate, and vibration transmission loss characteristics. Compared with the limitations of traditional manufacturing processes in terms of mechanical environmental adaptability, 4D printing technology can precisely control the stiffness gradient, deformation mode, and actuation response of materials through multi-material heterogeneous structure design and lattice programming arrangement. It is worth noting that although shape memory alloys, as typical self-healing materials, have demonstrated significant economic benefits and performance advantages in engineering applications, their phase transition kinetics behavior is highly sensitive to changes in the temperature field. When the ambient temperature undergoes transient fluctuations, the phase transition hysteresis characteristics of the material will lead to a hysteresis in the actuation response, which poses a severe challenge to the design of real-time control systems. The current research lacks quantitative evaluation of key parameters, such as the critical temperature of phase transition and the stability of thermomechanical cycling. The accumulation of relevant experimental data will provide important theoretical support for the future development of temperature compensation control algorithms.

Three-dimensional (3D) printing technology is an important technology for metamaterial manufacturing. In order to obtain a response to the outside world and improve its printed materials, 4D printing technology has been created. However, due to technical limitations and practical engineering applications, systematic analysis and adjustment have to be made. Compared to traditional building materials, the metamaterial cost dependent on printing technology is obviously higher, and the corresponding multi-physical field bearing capacity is relatively weak. Therefore, the main body is made of traditional building materials in practical engineering applications, and 3D-printed metamaterial patches are used in key areas. With the development of metamaterial research, single functionality cannot meet the actual needs of engineering, and versatility has become the mainstream of research; metamaterial versatility mainly depends on multi-material composites. In the process of printing multi-material, it is necessary to accurately control the nozzle switching; otherwise, there will be interface defects [117]. In addition, due to different shrinkage rates of different materials, it will lead to interlayer stress and deformation. Therefore, multi-material printability has become a key challenge for printing technology. It is worth noting that material compatibility should be fully considered in the process of metamaterial design. Due to material system limitations, it is often accompanied by some functional defects, such as environmental sensitivity and insufficient durability of the 4D printed materials [118]. Therefore, functional trade-offs and cross-scale coupling should be fully considered. At present, the main optimization strategies use topology optimization algorithms and bionic interfaces to balance performance and solve obvious performance defects. The field deployment directly affects the practical application of metamaterials in civil engineering, especially in the post-treatment process. For metal metamaterials, annealing is used to eliminate internal stress [28]. Polymer metamaterials use UV to cure the surface to achieve waterproof and anti-corrosion effects [119]. Post-treatment may account for 30~50% of the total manufacturing time and increase energy consumption. In addition, according to the analysis of many pilot-scale cases, the main problem existing in the actual application is the load problem: an additional 20~30% safety margin should be reserved beyond the theoretical value, and the scanner should be used to detect internal defects on-site. The future of 3D printing and 4D printing scale applications needs to overcome the impossible compatibility problem of scalability, material compatibility, and environmental robustness. At present, the dominant trend is to optimize this problem through modular design and hybrid manufacturing. The future should rely on new material development and cross-scale manufacturing technology.

### 5.2. Extraordinary Stiffness and Toughness

**Figure 19:**
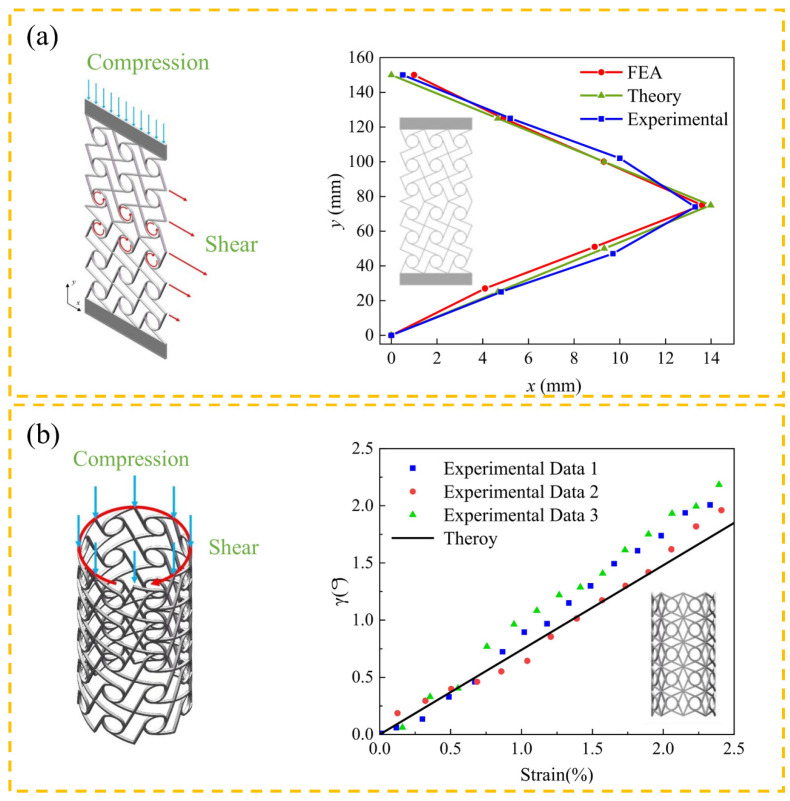
Mechanical behavior and experimental results of chiral metamaterials with different shapes: (**a**) structural design and compression–shear coupling deformation of two-dimensional chiral metamaterials; (**b**) structural design and compression–torsion coupling deformation of three-dimensional chiral metamaterials (*γ* is the torsion angle) [130].

Mechanical metamaterials, with their programmable topological configuration and multi-scale mechanical response characteristics, exhibit outstanding fracture toughness and tensile damage tolerance. At the same time, they achieve multi-functional integrated design, including multi-mode designs [20,121] and dynamic-static mechanical analysis [122]. The research team has carried out customized metamaterial design for typical engineering structures. Talebizadehsardari et al. [123] systematically revealed the influence mechanism of microstructure parameters such as small-scale coefficients, opening angles, and weight fractions on curved beams through parametric finite element simulation. It is worth noting that as a typical complex load condition, the asymmetric shear–stress distribution and section warpage effect generated by torsional load have long troubled engineering design. However, mechanical metamaterials can synchronously regulate the bent-torsional deformation mode through chiral symmetric breaking design, significantly improving the torsional collapse resistance performance of the structure [124]. Engineering practice shows that in the actual usage environment, structures often bear the synergistic effect of multi-axial stress rather than a single load condition. The multi-physics field coupling design based on metamaterials can effectively coordinate complex mechanical behaviors such as compression and shear coupling effects, thereby enhancing the stability margin of the structure under the composite load spectrum [125]. As shown in Figure 19, chiral isotropic isomers exhibit unique shear-induced anisotropy. Experiments on the prototype fabricated by 3D printing technology demonstrate that this structure can simultaneously suppress bending deflection and torsional deformation.

The current research focuses on the microstructure parametric design of mechanical metamaterials, aiming to achieve the synergistic improvement of the durability and multi-functional coupling characteristics of engineering structures throughout their life cycle through multi-objective optimization algorithms. Studies have shown that parameters such as the topological configuration of the cell, characteristic size, and material composition have a significant regulatory effect on mechanical indicators [35]. Through the damage tolerance design criterion, mechanical metamaterials can construct structures with gradient stiffness distribution. Their large deformation reversibility and multi-degree-of-freedom motion control ability can meet the adaptive requirements of engineering structures in various scenarios [126]. It is particularly necessary to point out that the high-strength and low-stiffness structure can achieve the regulation of stress wave propagation delay and damage accumulation threshold through the axial–rotational stiffness decoupling design, which has important engineering value for improving the anti-explosion and anti-impact performance of engineering protection structures [127]. Further research shows that the multi-level mesh metamaterials inspired by the principle of fractal self-similarity have improved crack propagation resistance and bending stiffness compared with traditional structures, effectively solving engineering problems such as deflection control of large-span structures [128]. In the field of advanced manufacturing technology, 4D printing has successfully fabricated active metamaterial structures with the function of adjusting environmental response stiffness. The load capacity of this type of structure is enhanced under environmental stimulation, and the absorption efficiency of impact energy is improved, demonstrating significant adaptability to multiple working conditions [129]. Although phased progress has been made in the stiffness and toughness co-design of metamaterials driven by digital twin models at present, there are still obvious deficiencies in the research on the fatigue crack initiation mechanism and the interface degradation behavior under cyclic loading in engineering applications. It is recommended to adopt the three-point bending fatigue test and carry out the reliability assessment based on the accelerated life test. Table 3 shows concrete durability evaluation methods and standards according to the American Society for Testing and Materials and the International Organization for Standardization, which can be used as a reference to evaluate the durability of metamaterials in engineering applications and predict their service life.

**Table 3:** Evaluation methods and standards for durability of concrete.

Test Type	Test Condition	Test Method	Assessment Criteria
Cycle fatigue	Pressure level 0.5 f_c_	Three-point bending or axial compression loading was applied at a frequency of 1 Hz until the specimen broke.	Stress–life curve, residual strength, stiffness degradation
Creep	Constant load 0.3 f_c_, 1000 days at 20 °C	A cylindrical specimen (Φ150 × 300 mm) was subjected to constant stress, and strain was continuously measured for 1000 days.	Creep coefficient
Cycle of freezing and thawing	300 freeze–thaw cycles	After immersion, the specimen was subjected to freezing and thawing cycles between −18 °C and 4 °C, and the mass loss and dynamic elastic modulus were tested every 50 cycles.	Mass loss and dynamic elastic modulus
Chloride ion penetration	90-day salt spray test	Immerse in 3% NaCl solution and periodically sample for chloride ion concentration profile.	Diffusion coefficient of chloride ion
Carbonization	CO_2_ concentration 20%, humidity 60%, 1 year	Under the condition of accelerated reaction test, carbonization was detected by phenolphthalein reagent.	Carbonation depth

### 5.3. Mechanical Response

**Figure 20:**
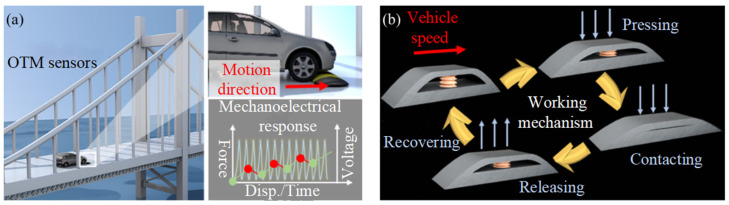
(**a**) The OTM is placed in the speed bump to automatically monitor the speed. (**b**) Working principle diagram of OTM in speed bump [134].

Mechanical metamaterials, with their multi-physics field coupling design and programmable intelligent response characteristics, provide a new technical path for the development of active adaptation systems in civil engineering structures. This type of metamaterial can achieve a perception–response closed-loop control of external excitation through piezoelectric/electromagnetic coupling effects and simultaneously complete the dual functions of energy capture and state monitoring [64]. The current mainstream energy-harvesting metamaterials mainly include triboelectric metamaterials, piezoelectric metamaterials, and thermoelectric metamaterials. The specific selection depends on the energy conversion principle, power density, conversion efficiency, and key constraints. Triboelectric metamaterials are periodically contacted and separated by different electronegative materials, causing charge transfer and electrostatic induction. The power density is 1~500 W/m^2^, and the actual conversion efficiency is 30~50%. Humidity greater than 60% will result in rapid decay of surface charges. Long-term friction results in low durability, and at low frequencies, output power is reduced, requiring frequency amplification structures [131]. Piezoelectric metamaterials use the stress–charge coupling effect to convert vibration into electrical energy, with a power density of 10~300 µW/cm^2^ and a conversion efficiency of 10~30%. Due to the working principle, it is only efficient in the resonance frequency range, and the commonly used materials are mostly brittle materials with low strength. When the frequency is lower than 50 Hz, the efficiency will be significantly reduced, requiring mass amplification strain. It is mostly used in building vibration energy recovery and self-energy wireless sensing [132]. Thermoelectric metamaterials are based on the Seebeck effect, generating potential differences by utilizing temperature gradients. The power density is 1 to 100 µW/cm^2^, and the conversion efficiency is 5% to 15%. Due to the working principle, stable temperature gradient, and easy-to-occur heat loss, high-cost constant thermal insulation materials are used. Thermal metamaterials are often used in industrial waste heat recovery [133]. In 2023, Jiao et al. [134] developed an origami tribo-metamaterial (OTM) for road speed bumps. This structure realizes the conversion of kinetic energy into electrical energy through the bistable folding configuration and simultaneously uses the deformation–voltage mapping relationship to invert the axle load and speed parameters of the vehicle (Figure 20). This marks the transition of piezoelectric intelligent paving systems from the laboratory to engineering practice, paving the way for the development of self-sensing and self-adaptive infrastructure. However, current research mostly focuses on the unit component scale, and in-depth exploration is still needed in the multi-scale integration and energy management optimization of the structural health monitoring system.

**Figure 21:**
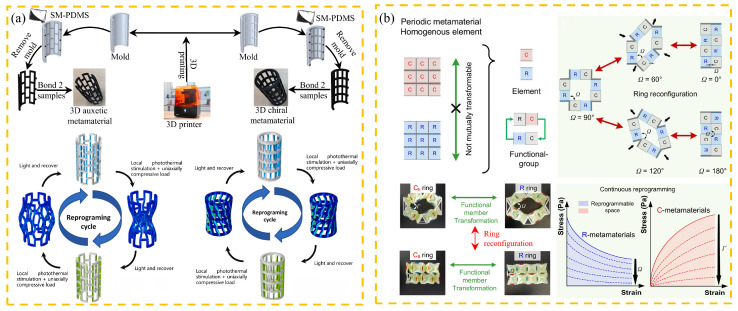
Examples of mechanical metamaterial programming design: (**a**) flexible mechanical metamaterials are reprogrammed using the light-induced SM effect (SM-PDMS is shape-memory polydimethylsiloxane) [126]; (**b**) mechanical metamaterial of origami reprogrammed by functional group transformation and programmable range (complete–elastic C or rigid–elastic R; Ω and Γ represent the angular relationship between adjacent elements; CII ring is a ring with a partially stretched or compressed functional group; and R ring is a ring with a full-length compressed functional group) [39].

The programmability of mechanical metamaterials marks the shift from passive loading to active adaptation of engineering materials, the core of which lies in the realization of dynamic regulation of mechanical properties and coordinated response of multiple physical fields through structure–material–function coupling design. The programmability of mechanical metamaterials stems from their periodic structural parametric design and the diversity of constituent materials, allowing adaptive structural design. Through parametric design of periodic structures, desired macroscopic mechanical behavior can be programmed, for example, negative Poisson ratio structures [135] and gradient stiffness designs [136]. Mechanical metamaterials are functionally integrated by multi-material systems, including active–passive material composites, and overall performance can be optimized by adjusting the properties of individual components. This programmability enables the materials to exhibit customized mechanical responses, both isotropic and anisotropic, and optimize their mechanical properties according to specific orientations. This adaptability to strain makes mechanical metamaterials highly versatile in a variety of engineering applications [38]. However, the design of stress transfer and energy dissipation at interfaces between different materials are key challenges. In recent years, programmability has emerged as a key research focus, intersecting with various facets of mechanical metamaterials. The overall implementation includes design methods and implementation paths, which include reverse design [137,138], thermal excitation mechanical response [24,139], and regulation of stiffness and toughness [39,41]. Reverse design abandons the limitations of traditional trial-and-error methods and quickly generates metamaterial topologies that meet target mechanical properties. Combining topology and genetic algorithms, performance coordination from microscopic elements to macroscopic structures is achieved. For example, Tian et al. [140] used neural networks to design lightweight seismic structures with specific Poisson ratios and energy absorption efficiencies. An illustrative example of a programmable mechanical metamaterial is shown in Figure 21, showcasing the dynamic capabilities of these advanced materials.

Programmable metamaterials and multifunctional integrated systems realize the trinity of engineering—self-sensing, self-energy, and self-regulation—and dynamic structural morphology control, improving the adaptability of structures. This is not only a concern in the field of civil engineering but also the focus of attention in various fields. At present, the engineering adaptive degree faces enormous challenges. Micro- to nano-scale metamaterials rely on 3D printing technology, resulting in high production costs. In engineering, many physical fields act on the component. The numerical solution of the thermal–mechanical–electric–magnetic coupling equation is inefficient, so it is necessary to develop a simulation platform accelerated by quantum computing. The future should focus on the optimization of multi-objective algorithms to solve trade-offs between mechanical performance, energy efficiency, and manufacturing cost. For example, for triboelectric speed bump optimization, Pareto optimal solutions between vehicle deceleration efficiency and energy collection should be found.

## 6. Conclusions and Prospects

The integration of metamaterials into civil engineering represents a transformative advancement in the design and functionality of infrastructure. Acoustic metamaterials have proven their efficacy in addressing noise pollution across a range of environments, from urban high-rises to roadways and public venues. By enabling superior sound isolation, these materials offer promising solutions for reducing noise pollution, improving quality of life, and meeting increasingly stringent acoustic standards in civil engineering. Mechanical metamaterials, on the other hand, provide unprecedented capabilities in terms of structural resilience. With unique attributes, such as shape memory, enhanced stiffness, and toughness, these materials have the potential to revolutionize the design of buildings, bridges, tunnels, and other civil infrastructure. The programmability of mechanical metamaterials further enhances their versatility, allowing for dynamic adjustments to accommodate varying mechanical demands, such as seismic forces, vibrations, and stress responses. This adaptability positions metamaterials as key enablers in the development of smart, responsive infrastructure.

Considering the environmental conditions, engineering cost, construction technology, theoretical innovation, and intelligence of civil engineering and based on the above conclusions, this review suggests that the following aspects can be strengthened in future research on the application of metamaterials in civil engineering:

(1) Enhanced durability and self-healing ability. Self-healing materials can repair damage and restore mechanical properties automatically after cracks or damage. However, their durability and environmental adaptability have become major obstacles to their development. Future research in this field should focus on optimizing and evaluating this property to generate more abundant data to support engineering applications. Self-healing mechanisms can be designed by combining microcapsules, microorganisms, self-healing materials, and metamaterial structures to develop new high-performance metamaterials, which can be compared and analyzed according to concrete performance tests.

(2) Cost-effective and scalable manufacturing methods. Three-dimensional (3D) and four-dimensional (4D) printing technologies are often hampered by high cost and scalability issues, which prevent metamaterials from being scaled in engineering. Three-dimensional printing technologies should improve printing speed, resolution, and the ability to print multiple materials via nozzle switching optimization, which can significantly reduce production costs and time. In addition, 4D printed structures can change shape or function in response to environmental stimuli but will lose effectiveness for complex environmental effects, requiring technical optimization and post-processing solutions to ensure structural practicality.

(3) Adaptive and smart infrastructure. Metamaterials have shown obvious potential in early programmable design, but their single performance adaptive embodiment ignores other performance evaluations and even sacrifices other key properties. Metamaterials are in the preliminary stage of programmability research, and most of them stay in the experimental stage. In the future, this field should focus on overall systematic design and analysis to truly realize practical engineering applications, such as using topology optimization algorithms to adjust design strategies and obtain optimal solutions for performance balance. Through systematic testing, more data can be obtained, and a data platform can be established to promote the practical research of programmable metamaterial integration engineering in the future.

(4) Energy harvesting and sustainability. Metamaterials with energy-harvesting capabilities are expected to improve the sustainability of civil engineering practices, but due to their working principles and narrow bandgaps, conversion efficiency is too low, and only some low-energy scenarios can be realized. Integration and bandgap widening of multiple metamaterials become key challenges. Multi-functional integrated system design should be carried out for scenarios, and broadband gaps should be expanded through topology optimization to improve energy conversion efficiency and realize passive sensing and monitoring integration.

In conclusion, although the wide application of metamaterials in civil engineering is still in the early stage, the current research focuses on band gap widening and topology optimization and lacks consideration and evaluation of overall performance. The most critical issues in the future are structural coupling, comprehensive design, data diversity, and the optimization of structure and manufacturing technology to reduce manufacturing costs and achieve scale. Finally, metamaterial programmability research will be a long-term concern in the future, with design complexity and high manufacturing technology requirements becoming major challenges. As research and development continue to push the limits of material science, we can expect to see more efficient, sustainable, and intelligent solutions that enhance the resilience and functionality of built environments.

## Figures and Tables

**Table 1:** Comparison of properties between traditional materials and metamaterials.

Property	Traditional Materials	Metamaterials
Noise reduction	Wide-band sound insulation (depends on the density and structure of the material)	Sound insulation in specific frequency bands and even the “stealth” of sound waves can be realized [8,10].
Heat insulation	Thermal conductivity is fixed (depends on the thickness and type of material).	Modulation of thermal conductivity for special effects of thermal insulation or heat absorption (e.g., thermal “stealth”) [14,16,32]
Lightweight and high-strength	There is a trade-off between strength and weight.	Balance of high strength and low density (depending on nanoscale structure design) [33]
Corrosion resistance	Embalming is required.	Resist corrosion and aging (design-dependent) [34,35]
Self-healing	Manual repair is required.	Self-healing properties (depending on material properties) [22,36,37]
Durability	The technology is mature and dominant.	Exploratory phase (innovative)
Programmability	-	High programmability (depending on the precise design of the microstructure) [38,39,40,41]
Energy harvesting and response	-	Active and efficient energy harvesting and conversion and fast and flexible energy response [12,38,42,43,44]
Environmental impact	The environmental impact is more obvious.	Low energy consumption and environmentally friendly [45,46,47]
Sustainability	Poor sustainability (high consumption in the production process and difficulty in recycling)	Sustainable (great potential and more opportunities for innovation) [47]

## Data Availability

No new data were created or analyzed in this study. Data sharing is not applicable.
